# Distribution Patterns (7B Rule) and Characteristics of Large Congenital Melanocytic Nevi: A Retrospective Cohort Study in China

**DOI:** 10.3389/fmed.2021.637857

**Published:** 2021-02-19

**Authors:** Huijing Wang, Wei Wang, Jun Lu, Yihui Gu, Xiwei Cui, Chengjiang Wei, Jieyi Ren, Bin Gu, Zhichao Wang, Qingfeng Li

**Affiliations:** ^1^Department of Plastic and Reconstructive Surgery, Shanghai Ninth People's Hospital, Shanghai Jiao Tong University School of Medicine, Shanghai, China; ^2^Division of Epidemiology and Biostatistics, School of Public Health, University of Illinois at Chicago, Chicago, IL, United States

**Keywords:** Chinese, retrospective cohort study, malignancy rate, 7B rule, distribution pattern, pathology, large congenital melanocytic nevus

## Abstract

Large congenital melanocytic nevus has a high risk of malignancy. However, few studies have summarized its characteristics, treatments, outcomes and malignancy incidence in Chinese patients. This paper reviews a retrospective cohort study evaluating 1,171 patients from Shanghai Ninth People's Hospital between 1 January 1989 and 31 August 2019 using electronic medical records and phone calls to collect clinical and pathological data in which 133 patients were diagnosed with a large congenital melanocytic nevus. Three patients relapsed, and none developed melanoma among the qualified patients. Besides, a new “7B” rule for distribution patterns of large congenital melanocytic nevi was proposed, including bonce, bolero, back, bathing trunk, breast/belly, body extremity, and body. The most common distribution pattern of large congenital melanocytic nevi was bonce, and all blue nevi distributed as bonce. Statistical analysis showed a significant difference (*P* = 0.0249) in the “7B” patterns between the melanocytic nevus and the neuronevus. In conclusion, the malignancy rate of large congenital melanocytic nevi is much lower in China than in other regions and people of other races. The pathology of large congenital melanocytic nevus may decide its “7B” distribution pattern.

## Introduction

Large congenital melanocytic nevus (LCMN) is a rare disease that occurs in a range of ~1 in 20,000 to 1 in 500,000 newborns (1, 2). It is acknowledged that the most common somatic mutation in congenital melanocytic nevi (CMNs) is *NRAS* mutation, but there are potential alternative mechanisms. One recent research showed that *NRAS* mutation existed only in 57.1% of large/giant CMNs. The remains had gene fusions or point mutations involving other genes, such as *BRAF* (3). Once born with LCMN, the patients' appearance and life quality are significantly affected. Extensive research has demonstrated that LCMN conveys a high risk for melanoma formation. The largest meta-analysis of LCMN by Vourc'h-Jourdain et al. (4) reported an overall risk of malignancy of ~2%. Among Caucasian people with LCMNs, the lifetime risk of developing both cutaneous and non-cutaneous melanoma is 4.5–10% (5). Additionally, Price et al. (6) demonstrated that ~5% of LCMNs transforms into melanoma, and half of these transformations occur early in life. By contrast, only around 2.3% of the general population will be diagnosed with melanoma of the skin during their lifetime, based on the data collected by the U.S. National Cancer Institute from 2015 to 2017.

The incidence rate of melanoma in Chinese patients with LCMNs remains unclear since only a few studies have investigated LCMNs and the risk of melanoma regarding Asian patients. A nationwide retrospective study performed in Korea found that melanoma developed in 3 of 131 LCMN patients, indicating the incidence rate was 2.29% (7). A systematic review showed that Southeast Asian patients with LCMNs, including 29 Chinese, 6 Malay, 1 Indian, and 3 Caucasian patients, did not develop any form of malignancy, with the longest follow-up extending to 38 years (8). Briefly, the above results all illustrate that the incidence of malignancy in Asians is significantly different from that in the Caucasian population (9).

Recently, Martins et al. (10) summarized and proposed a “6B” rule concerning the patterns of distribution of giant congenital melanocytic nevi (GCMNs), referring to the bolero, back, bathing trunk, breast/belly, body extremities, and body. However, the rule does not involve the region of head and face, where LCMNs also occur and create even more disease burden for these patients.

We supposed that the rate of malignancy and characteristics of LCMNs in China are different from those in other regions and people of other races. Therefore, a comprehensive study to analyze the characteristics, treatments, outcomes and risk of melanoma formation among Chinese LCMN patients is highly needed.

## Methods

### Patients

The clinical and pathological data were collected from LCMN patients registered and treated at Shanghai Ninth People's Hospital, Shanghai Jiao Tong University School of Medicine from 1/1/1989 to 8/31/2019. A total of 1,171 patients with diverse nevus types were screened.

The inclusion criterion was that the size of CMN expected by adulthood, also known as the projected adult size (PAS), would be larger than 20 cm and smaller than 40 cm at its greatest diameter (11). For adults, the size of nevi was measured by physical examination whereas PAS graphs were used for children. This single-center database contained individuals' personal information, medical history and all reports from clinical tests.

This study was approved by the institutional ethical committee of Shanghai Ninth People's Hospital, Shanghai Jiao Tong University School of Medicine (SH9H-2019-T163-2, September 30, 2019). Informed consents were obtained from all individual participants included in the study.

### Clinical Information

The details concerning clinical and phenotypes features of LCMNs were acquired through the electronic clinical database and telephone survey.

According to Krengel's recommendation, the morphological characteristics include color heterogeneity, surface rugosity, hypertrichosis, and the presence of dermal or subcutaneous nodules (11). There was a need to modify and adjust the standard to make the description more suitable for our research. Thus, we focused on whether the LCMN had the following features: evolving from the surface, dark in color, wrinkly and/or hairy surface, presenting nodules, hard of texture, painful to touch and/or itchy. Each phenotype was divided into the subgroup “Yes” or “No.” The subgroups of nevi were decided through medical records and patients' answers. Regarding the anatomic localizations of LCMNs on the skin surface of patients, we used specific terms, including bonce (head and facial region), bolero, back, bathing trunk, breast/belly, body extremity and body to generalize the distribution patterns. Specifically, “Bonce” is referred to head and facial regions. “Bolero” is mainly on the upper back including the neck. “Back” is usually in round shape and does not involve buttocks or shoulders. “Bathing trunk” mainly involves the genital region and buttocks but excluding shoulders and neck. “Breast/belly” only distributes on chest or abdomen and no overlap with bolero or bathing trunk. “Body extremity” only locates on extremity and excludes of shoulders and genital region. “Body” is a pattern combines bolero and bathing trunk, which affects almost the entire body.

We also collected information about whether neurological symptoms, comorbidities, or relapses were present. Surgical excision procedures, such as skin graft, hemorrhoidectomy, flap graft and skin dermabrasion, and pathological diagnoses were also performed. Relapsed patients were carefully reviewed.

Additionally, typical histopathological characteristics were listed throughout the clinicopathological diagnosis reports—for example, which tissue was hyperplasic or which layer had invaded melanocytes. The pathology of LCMN was classified as the junctional nevus, the intradermal nevus, the compound nevus, the blue nevus or the neuronevus. The junctional nevus, intradermal nevus and compound nevus were also collectively known as the melanocytic nevus.

### Statistical Analysis

Descriptive statistics were presented using counts and percentages. *Post-hoc* tests following Fisher's exact test were used to determine which paired comparisons were significantly different if an association exists. The Benjamini-Hochberg procedure was used to correct *P*-values during the multiple comparison. Statistical analysis was performed using R 3.5.3, Statistical Software (Foundation for Statistical Computing, Vienna, Austria).

We hypothesized that phenotypes and distribution patterns of LCMNs among the melanocytic nevus, blue nevus and neuronevus groups were significantly different. The significance level was defined as a *P*-value of <0.05.

## Results

### Demographic and Clinical Characteristics of LCMNs

One hundred thirty-three patients were diagnosed with LCMNs between 1/1/1989 and 8/31/2019 and included 58 (43.6%) males and 75 (56.4%) females. The median length of follow-up time was 43 months, and the longest follow-up time was 360 months. Specific statistics on the follow-up length are shown in Table 1.

**Table 1:** Baseline characteristics of large congenital melanocytic nevi.

	**No. (%)**
Sample size	133 (100.0)
Male	58 (43.6)
Female	75 (56.4)
**Length of follow-up, Y**	
0~1	6 (4.5)
1~2	30 (22.6)
2~3	22 (16.5)
3~4	15 (11.3)
4~5	14 (10.5)
5~6	10 (7.5)
6~7	10 (7.5)
7~8	8 (6.0)
8~9	6 (4.5)
9~10	3 (2.3)
>10	9 (6.8)
**Distribution pattern**	
Bonce	55 (41.3)
Bolero	34 (25.5)
Back	11 (8.3)
Bathing trunk	15 (11.3)
Breast/belly	4 (3.0)
Body extremity	9 (6.8)
Body	5 (3.8)
**Neurological symptom**	
Myasthenia gravis	1 (0.7)
**Surgical procedure**	
Skin graft	52 (39.1)
Hemorrhoidectomy	11 (8.3)
Flap graft	64 (48.1)
Skin dermabrasion	2 (1.5)
Surveillance	4 (3.0)
**Comorbid**	
Vitiligo	1 (0.7)
Neurofibromatosis 1	1 (0.7)

**Figure 1:**
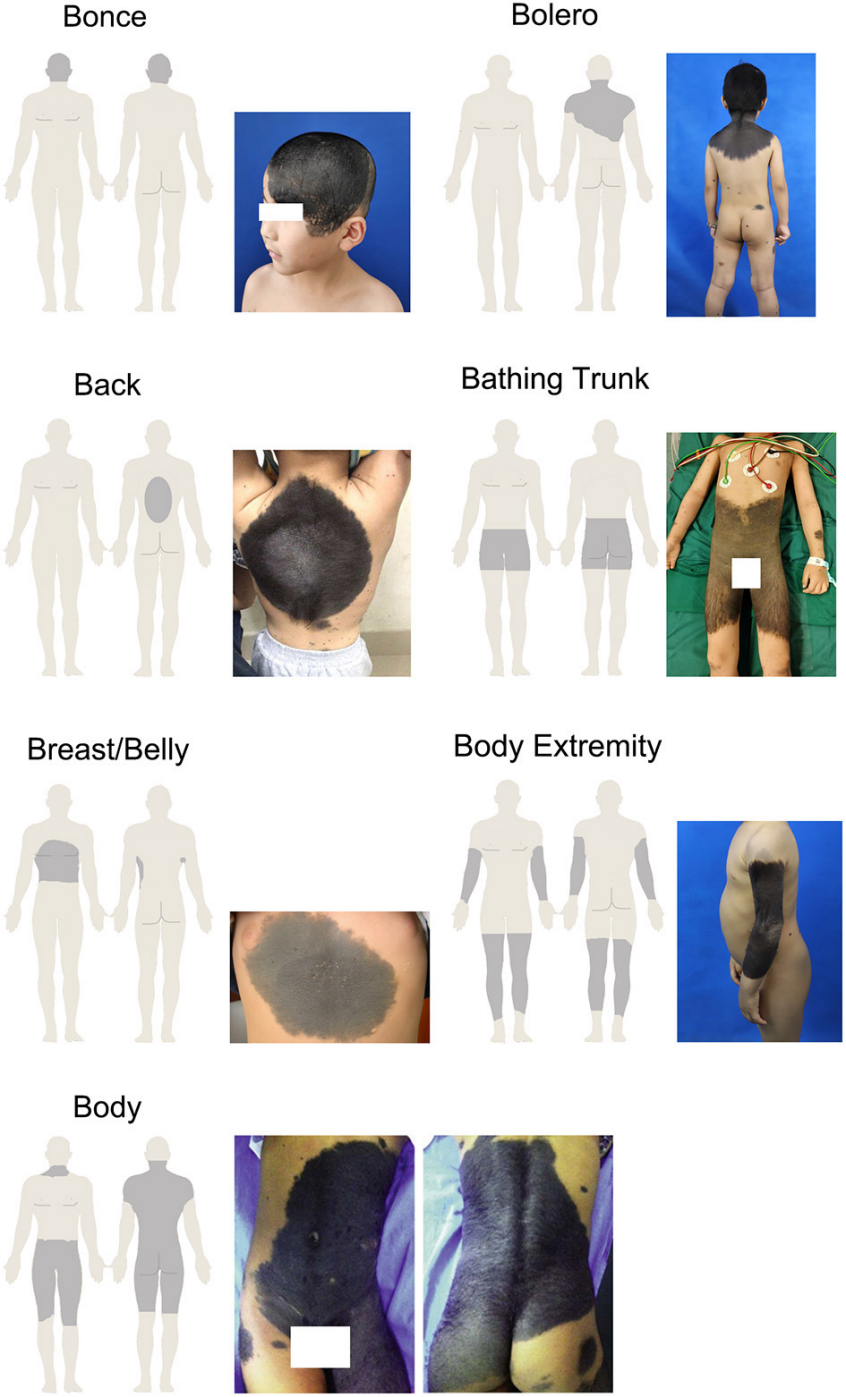
Distribution patterns of large congenital melanocytic nevi—“7B” rule.

Table 1 also shows that distributions of LCMNs in the 133 patients according to the new “7B” rule (Figure 1) modified from the “6B” rule. The new “7B rule” consists of 7 patterns: bonce (41.3%), bolero (25.5%), back (8.3%), bathing trunk (11.3%), breast/belly (3.0%), body extremity (6.8%), and body (3.8%). Myasthenia gravis was present as a neurological symptom in one (0.7%) patient. Comorbid diseases were also evaluated. Two patients had comorbid vitiligo (0.7%) and neurofibromatosis type 1 (0.7%). Skin graft (39.1%), hemorrhoidectomy (8.3%), flap graft (48.1%) and skin dermabrasion (1.5%) were performed for patients to remove nevi.

### Histopathological Findings of LCMNs

**Figure 2:**
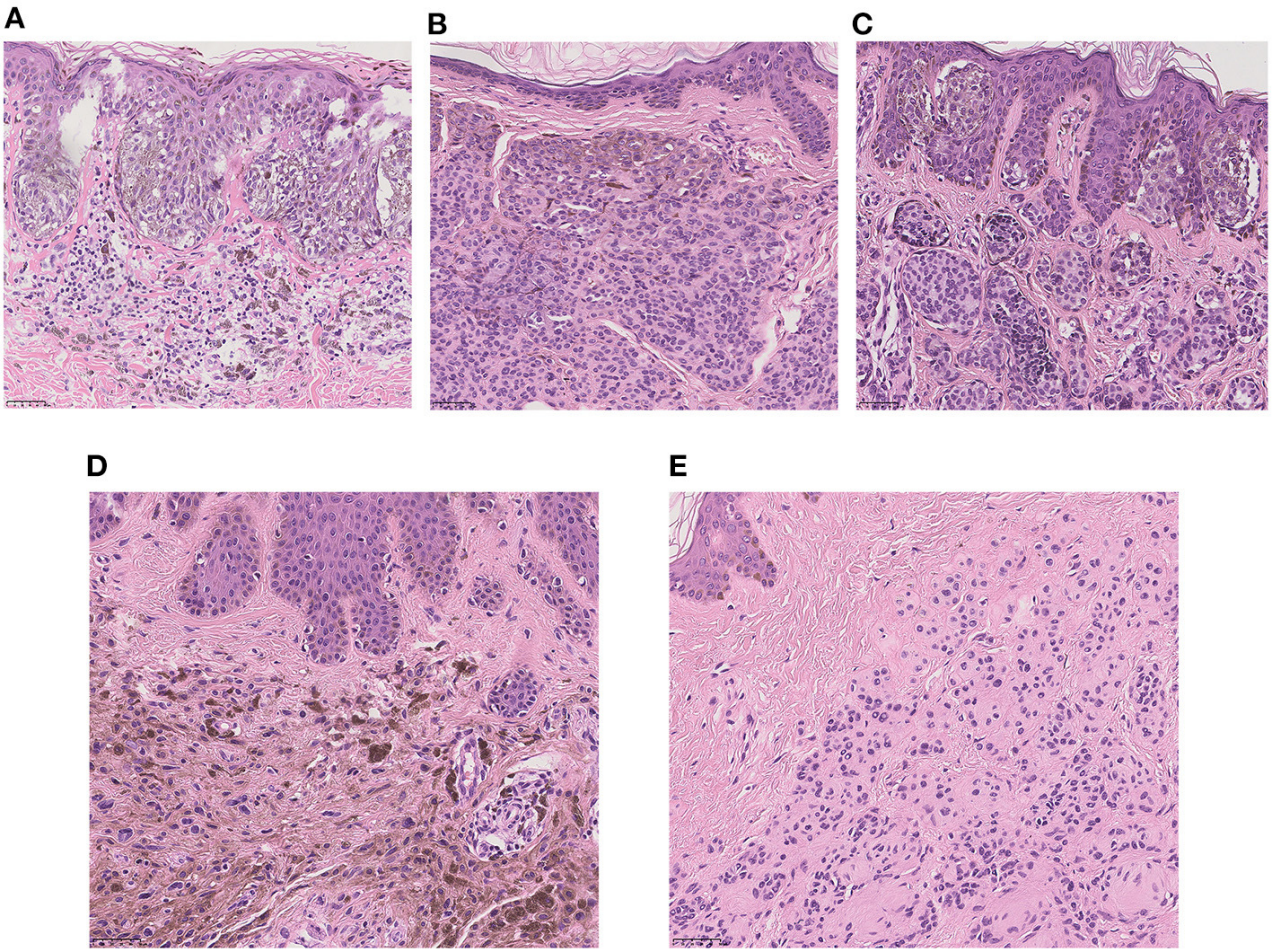
High-power view of the typical histopathology of LCMNs (H&E stain, ×20). **(A)** Junctional nevus. **(B)** Intradermal nevus. **(C)** Compound nevus. **(D)** Blue nevus. **(E)** Neuronevus.

Five pathological types were diagnosed among the 123 patients, and the pathological records of the rest of 10 (7.5%) patients were missing. The categories of pathology were as follows: the junctional nevus (2.3%), the intradermal nevus (24.0%), the compound nevus (56.4%), the blue nevus (2.3%) and the neuronevus (7.5%) (Figure 2). Only 93 of these 123 patients had a further detailed description of their histopathology; the remainder only had a confirmed diagnosis. We also have observed some typical histopathological features of LCMNs. One lesion may have multiple kinds of histopathological features simultaneously, such as collagenization and nodularization. Furthermore, 47 lesions had invaded to adipose tissue, and three lesions invade to striated muscle. These melanocytes infiltrated deeper and were harder to be removed by surgery.

Table 2 shows the eight phenotypes of LCMNs among the five main pathologies. Almost all LCMN cases were dark (*N* = 126; 94.7%) and hairy (*N* = 127; 95.5%). Another common phenotype was evolving, which was reported for 65.4% of all patients. Very few patients (*N* = 2; 1.5%) felt pain.

**Table 2:** Frequencies and percentage of different phenotypes of different pathological large congenital melanocytic nevi.

	**Evolving**	**Dark**	**Wrinkly**	**Hairy**	**Nodule**	**Hard**	**Painful**	**Itched**	**No. (%)**
Junctional nevus	2	3	3	3	1	0	0	1	3 (2.3)
Intradermal nevus	19	31	23	31	17	6	1	3	32 (24.0)
Compound nevus	49	71	58	70	42	13	0	11	75 (56.4)
Blue nevus	2	3	2	3	2	0	0	0	3 (2.3)
Neuronevus	9	9	8	10	8	1	1	3	10 (7.5)
Unknown	6	9	8	10	3	2	0	2	10 (7.5)
No. (%) in total patients	87 (65.4)	126 (94.7)	102 (76.7)	127 (95.5)	73 (54.9)	22 (16.5)	2 (1.5)	20 (15.0)	133 (100.0)

### Treatment Outcomes of LCMNs

LCMNs relapse occurred in 3 (2.3%) of the 133 people after surgery (Table 3), while the others remained stable. Notably, all of the relapsed patients had evolving, wrinkly, hairy LCMNs with nodules before surgery. However, each had undergone different operations for more than once. At the first stage, one had a skin graft, one had undergone hemorrhoidectomy, and one had a flap graft. The chance of relapse of each procedure equals the number of relapsed patients divided by the number of patients accepting this surgical procedure. We speculated that the chance of relapse of skin graft, hemorrhoidectomy and flap graft was 1.9, 9.0, and 1.6%, respectively. The nevus cells of two patients had invaded the fat layer, but the detailed histopathology of another patient remained unknown due to data loss.

**Table 3:** Clinical and pathological information about relapsed patients.

	**No. (%)**
Relapsed patients	3 (2.3)
**Gender**	
Male	1 (1.7)
Female	2 (2.6)
**Distribution pattern**	
Bonce	1 (1.8)
Bathing Trunk	2 (13.0)
**Phenotype**	
Evolving	3 (3.4)
Dark	2 (1.6)
Wrinkly	3 (2.9)
Hairy	3 (2.4)
Nodule	3 (4.1)
Hard	1 (4.5)
Painful	0 (0)
Itched	1 (5.0)
**Surgical procedure**	
Skin graft	1 (1.9)
Hemorrhoidectomy	1 (9.0)
Flap graft	1 (1.6)
**Pathology**	
Intradermal nevus	1 (3.1)
Compound nevus	1 (1.3)
Neuronevus	1 (10.0)

### Melanoma Formation in LCMN Patients and Systematic Review of Published Studies

After such an extended follow-up, not a single case in our cohort developed melanoma. By contrast, foreign research over the latest 40 years has shown different results (Table 4). The range of the incidence rate of LCMNs developing into melanoma varied from 0 to 8.52%. According to Hale, the rate was particularly high and reached 48.78% (20). However, 18 of the 22 studies were conducted only in western countries, and only a few included non-white people.

**Table 4:** Studies of large congenital melanocytic nevi included in the systematic review.

**References, country**	**No. of LCMN patients**	**Race or nationality**	**No. of patients developed MM**	**Crude incidence of MM in LCMNs**
Lorentzen et al. (12), Danmark	151	Danish	3	1.99% (4.6% lifetimes)
Quaba and Wallace (13), UK	39	Caucasian	2	8.52% (first 15 years of life)
Gari et al. (14), America	54	American	1	1.85%
Ruiz-Maldonado et al. (15), Mexico	80	Mexico	4	5.00%
Egan et al. (16), America	46	American	2	4.35%
Foster et al. (17), America	46	American	0	0
Berg and Lindelöf (18), Sweden	146	Caucasian	2	Unknown
Ka et al. (19), Sweden	397	Worldwide	0	0
Hale et al. (20), America	205	American	10	48.78%
Bett (21, 22), America	991	1 African, 1 Hispanic, 1 Japanese, others were worldwide	17	1.71%
Zaal et al. (23), Netherland	320	Caucasian	4	1.25%
Yuin-Chew et al. (8), Singapore	39	29 Chinese, 6 Malay, 1 Indian, and 3 Caucasian	0	0
Vito et al. (24), Italy	31	Italian	0	0
Warner et al. (25), America	40	American	0	0
Fernandes et al. (26), Brazil	5	4 white and 1 non-white	0	0
Kinsler et al. (27), UK	50	Caucasian	0	0
Turkmen et al. (28), Turkey	26	Turku	2	7.69%
Chen et al. (29), Sydney	31	Australian	0	0
Yun et al. (7), Korea	131	Yellow	3	2.29%
Kinsler et al. (30), UK	448	Caucasian	10	2.23%
Viana et al. (31), Brazil	57	South-American	2	3.51%
Fahradyan et al. (32), America	14	American	0	0

*LCMN, large congenital melanocytic nevus; MM, melanocytic melanoma*.

### Relationship Between Pathologies and “7B” of LCMNs

**Figure 3:**
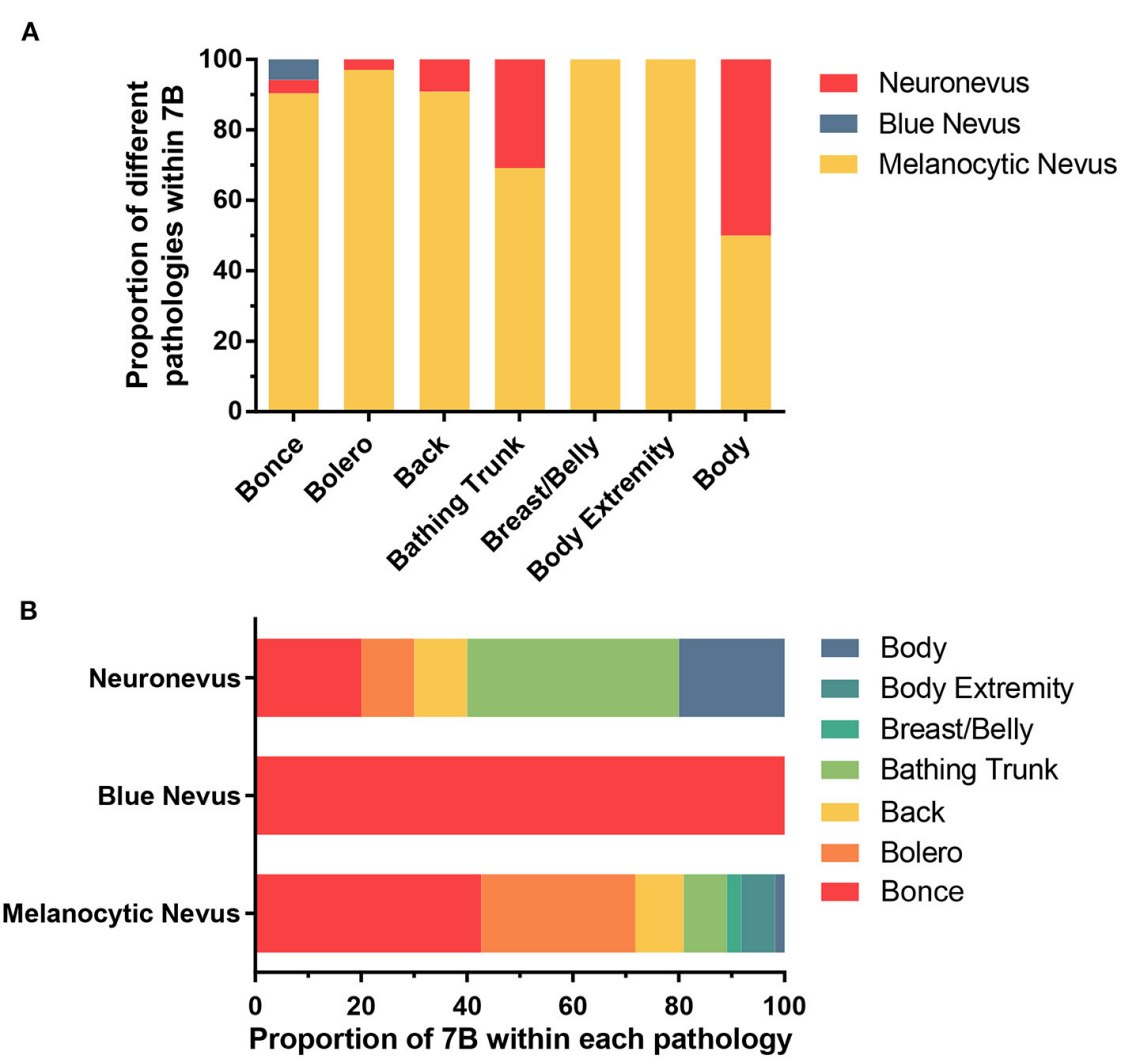
**(A)** Showing the proportion of three pathological types of LCMNs in each distribution pattern according to the “7B” rule. **(B)** Showing the proportion of each distribution pattern classified by the “7B” rule in different pathological types of LCMNs.

By grouping the junctional nevus, intradermal nevus, and compound nevus together as the melanocytic nevus, we formed three populations—melanocytic nevus, blue nevus and neuronevus (Table 5). Statistical analysis using Fisher's exact test revealed a significant result (*P* = 0.0295; α = 0.05) of distribution patterns among three kinds of pathological types. Furthermore, the difference between “7B” distribution patterns of the melanocytic nevus and the neuronevus was significant (*P* = 0.0249; α = 0.05). Thus, the melanocytic nevus and the neuronevus tend to occur in different distribution patterns on the body. In Figure 3A, the melanocytic nevus had the major proportion in every pattern except the “body,” which consisted of 50% the melanocytic nevus and 50% the neuronevus. Figure 3B shows us that all blue nevi in our cohort distributed as bonce, though there were only three cases in total.

**Table 5:** Pathology × distribution pattern cross tabulation.

		**Distribution pattern**							
	**No. (%)**	**Bonce**	**Bolero**	**Back**	**Bathing trunk**	**Breast/belly**	**Body extremity**	**Body**	**Total**
Pathology	Melanocytic nevus	47 (42.7)	32 (29.1)	10 (9.1)	9 (8.2)	3 (2.7)	7 (6.4)	2 (1.8)	110 (100.0)
	Blue nevus	3 (100.0)	0	0	0	0	0	0	3 (100.0)
	Neuronevus	2 (20.0)	1 (10.0)	1 (10.0)	4 (40.0)	0	0	2 (20.0)	10 (100.0)
	Total	52 (42.3)	33 (26.8)	11 (8.9)	13 (10.6)	3 (2.4)	7 (5.7)	4 (3.3)	123 (100.0)

## Discussion

Many types of CMNs have been identified. Among them, the LCMN is a rare and benign type of CMNs. LCMNs affect patients' appearance and self-esteem significantly. LCMNs have been defined in many ways and aspects. Zaal et al. (33) had performed a Medline literature search and summarized different clinical classifications of large/giant CMNs. Krengel et al. (11) proposed a new standard to define LCMNs (L1: 20–30 cm; L2: 30–40 cm). We adopted the new standard but did not distinguish L1 from L2 because of the unclear clinical records of some patients who registered in the system at early stages.

LCMNs can be located anywhere on the body and presented with various shapes. The descriptions of LCMN phenotypes are important because of their large sizes and wild heterogeneity. Some oncogenic mutations were only detected in a particular area of LCMNs, which indicates that different lesion areas may have different molecular characterization (3). Vanessa et al. proposed the “6B” rule to classify GCMNs in different locations into one certain pattern and suggested “bathing trunk” as the most frequently observed pattern (10). However, no specific description exists for patients with GCMNs on the head and/or facial regions in the “6B” rule. In our study, we created the “7B” rule based on the “6B” rule and discovered that the most frequent distribution of LCMNs was bonce. LCMNs that manifests in the region of the head and face have much worse effects on patients' appearances and lives. A recent study of quality of life (QoL) in children and adolescents with CMNs reflected that young patients experienced emotional impairment and felt shame if the CMNs were exposed to the public (34). We used Skindex-29, a widely acknowledged questionnaire for health-related quality of life (HRQoL) assessment, to evaluate the HRQoL of 19 patients in our cohort and obtain a consistent result. Reasonably, patients with congenital lesions on visible body parts, such as the face and hands, would visit the hospital and seek a cure more eagerly, explaining that bonce was most frequently observed in our cohort and caused admission rate bias.

Neurological symptoms rarely occurred in our patients, only one had myasthenia gravis. No correlation was found between LCMNs and myasthenia gravis, although a few nevi were observed that invaded striated muscle histopathologically in our study. Additionally, one case report mentioned that a 21-year-old male patient with myasthenia gravis had eruptive melanocytic nevi when treated with azathioprine and dexamethasone (35). In our study, two patients with LCMNs had concomitant vitiligo and neurofibromatosis 1. Two patients also had vitiligo in a retrospective study in Korea (7). Some clinical evidence revealed the relevance of CMN in vitiligo or NF1. Van Geel et al. (36) concluded that CMN could affect the age of onset of vitiligo and trigger the development of halo nevi in patients with vitiligo. Another report presented a rare case of an association between LCMNs, halo nevus and vitiligo (37). Histological results presented both nevus cell nests and neural elements located in the dermis in some cases (38, 39), demonstrating that neurofibromatosis and large pigmented nevus have a common origin (40). In addition, a 21-year-old Korean girl with a large congenital nevocytic nevus had both neurotization and the onset of vitiligo (41). The above cases reflect a certain relationship between congenital nevus and vitiligo or NF1, which requires further study.

Usually, the histopathology of the nevus is classified according to the location and growth pattern of melanocytic cells. The junctional nevus comprises melanocytes located at the junction of the epidermis and dermis, the intradermal nevus comprises melanocytes limited in the dermis, and the compound nevus comprises the junctional nevus and the intradermal nevus. Currently, the blue nevus represents dermal arrest in the embryonal migration of neural crest melanocytes that fail to reach the epidermis, causing the pigment to appear deeper in the skin than in ordinary nevi. Melanocytes of the neuronevus is also located intradermally and have typical nerve-like growth pattern. In our study, most LCMN cases were melanocytic nevi, and 68.1% of them were compound nevi. The blue nevus occurred the least frequently. The result was reasonable because conventional melanocytic nevus is the most common nevus pathological type, and the blue nevus is not commonly acquired, with a 3–5% prevalence in adults in the United States (42).

The histopathological features of LCMNs were not isolated but overlapped. Additionally, LCMNs invade into adipose or striated muscle tissue, indicating that LCMNs have great heterogeneity and diversity.

Only three patients relapsed. We assumed the current relapsed nevi were derived from original lesions because all relapsed LCMNs had invaded the fat layer. Thus, some melanocytes might have infiltrated too deeply to be removed entirely by surgery.

With approximately the same number of patients as the Korean cohort, we found no malignant cases while there were 3 in them. On the one hand, our data were only collected from a single center, yet researchers pooled data from 40 university hospitals in Korea, representing a much more comprehensive range of cases than ours. On the other hand, this result implies that the risk of LCMNs becoming melanoma differs greatly from region to region as well as from race to race. Although the molecular mechanisms of the formation and development of LCMNs still remain unclear, they should be taken into consideration. Races, food, climates and all the environment around patients can potentially influence the gene expression and cause mutations in LCMNs. The result reminds us the mechanisms behind this disease could be complicated and related to multiple factors. It also suggests that the risk of LCMNs transforming into melanoma is extremely low among Chinese people. Thus, it would be inaccurate if we directly use the estimated risk in other races to predict malignancy in Chinese or Asian people.

Krengel et al. (10) summarized the characteristics of each CMN size category and marked the phenotypes as three degrees from “0” to “2.” Although standard markers defined the degree, it is still confusing and almost impossible for our patients to describe their nevus type precisely through phone calls. To solve this problem, we extended and adjusted the content of the study by adding four additional phenotypes of lesions but simplified subgroup to: “yes” or “no.” This standard led to a much easier and faster evaluation of LCMNs.

To our best knowledge, our study is the first to investigate the relevance between pathologies and distribution patterns of nevi. According to pathological classification, the junctional nevus, intradermal nevus and compound nevus are considered the melanocytic nevus. The significant result among the melanocytic nevus, blue nevus and neuronevus, as well as the significant result between the melanocytic nevus and neuronevus, proved that the pathology of the LCMN is correlated with its pattern of distribution. The pathology and distribution correlation was not clarified mainly due to the inadequate statistical sample size. After collecting and analyzing a sufficient number of cases, we hope to figure out whether its pathology decides the distribution pattern of the LCMN. If so, we can infer the pathological type of the LCMN through its pattern before acquiring the exact pathological diagnosis by invasive biopsy.

In conclusion, 133 Chinese patients with LCMNs were carefully analyzed and some meaningful results were obtained. We proposed a brand-new term, “?once,” for the “7B” rule to describe distributions of LCMNs more comprehensively. Moreover, the specific distribution pattern may imply a potential pathology of the LCMN. Most importantly, we demonstrated the remarkably low incidence of melanoma arising from LCMNs in China, indicating the low risk of malignancy. We are also aware that the rate of malignancy in these patients may still be higher than that in the general population and further studies would be required to validate this conclusion. Additionally, treatments should be re-evaluated and the necessity of surgery should be reconsidered.

Although the present study is the largest and longest retrospective LCMN cohort in Asia, filling a huge gap in this field, there are still limitations. Firstly, this was a single-center analysis, which could miss multiple eligible patients across China. Secondly, many medical records by handwriting were unavailable until the electronic clinical database was created, causing a significant loss which could affect the study's accuracy. Although the follow-up time in our research is longer than many other retrospective cohort studies and the longest follow-up time is 360 months, 65.4% of patients in our cohort were followed up within 5 years and the median length of follow-up time was 43 months. The development of melanoma may require more time. We are supposed to follow up with these patients continually and expand our cohort to find out if there will be different results. Last but not least, the lack of magnetic resonance imaging (MRI) examination for each patient's lesion made us unable to see the direct structure of LCMN, which was also important.

We hope our findings not only help clinical doctors assess patient's condition and guide the treatment plan for the disease, but provide useful information and new ideas to inspire researchers for future investigations.

## Data Availability Statement

The raw data supporting the conclusions of this article will be made available by the authors, without undue reservation.

## Ethics Statement

The studies involving human participants were reviewed and approved by Shanghai Ninth People's Hospital, Shanghai Jiao Tong University School of Medicine. Written informed consent to participate in this study was provided by the participants' legal guardian/next of kin. Written informed consent was obtained from the minor (s)' legal guardian/next of kin for the publication of any potentially identifiable images or data included in this article.

## Author Contributions

QL and ZW contributed to the conception and design of the work. HW, WW, JL, and YG collected clinical information. HW and JL wrote the manuscript. JL performed data analysis. WW and YG contributed remarkably to interpretation of data. XC, CW, JR, and BG helped with constructive discussions. All authors substantively revised the work and have approved the submitted version.

## Conflict of Interest

The authors declare that the research was conducted in the absence of any commercial or financial relationships that could be construed as a potential conflict of interest.
