# Supplementary material for: Distribution Patterns (7B Rule) and Characteristics of Large Congenital Melanocytic Nevi: A Retrospective Cohort Study in China
Source: Front Med (Lausanne). 2021 Feb 19;8:637857. doi: 10.3389/fmed.2021.637857 (PMC7933508; doi:10.3389/fmed.2021.637857)
Supplement: Supplementary file 1 [file Table_1.DOCX]

**Phone Question Set**

Interviewer: ; Date/Time: ;

Hello, this is LCMN research group from Department of Plastic and Reconstructive Surgery, Shanghai Ninth People’s Hospital. We are currently conducting a follow-up survey in patients with large congenital melanocytic nevus.

Here are some questions for you. This might take you 5-10 minutes.

Thank you very much for your kind cooperation.

1. What is your name? *

_________________________________

**Section I. Distribution of Large Congenital Melanocytic Nevus**

2. Choose the distribution of your large congenital melanocytic nevus. *

| ○Back: On the back, often round shaped, no involvement of buttocks or shoulders. |
| --- |
| ○Bathing Trunk: Mainly genital region and buttocks. Does not reach shoulders or neck. |
| ○Body: Bolero and bathing trunk jointly affecting almost the whole body. |
| ○Bolero: Mainly involving upper back including neck. |
| ○Breast/Belly: Isolated to chest or abdomen. Not mixed with bolero or bathing trunk. |
| ○Body Extremity: Isolated to extremity. No involvement of shoulders or genital region. |
| ○Βέλος: On the face and head. |

**Section II. Phenotypes of Large Congenital Melanocytic Nevus**

3. Was your large congenital melanocytic nevus growing? *

| ○Yes |
| --- |
| ○No |

4. Was your large congenital melanocytic nevus heterogeneous in color? *

| ○Yes |
| --- |
| ○No |

5. Was your large congenital melanocytic nevus wrinkly on the surface? *

| ○Yes |
| --- |
| ○No |

6. Whether your large congenital melanocytic nevus hairy on the surface? *

| ○Yes |
| --- |
| ○No |

7. Did the large congenital melanocytic nevus feel hard? *

| ○Yes |
| --- |
| ○No |

8. Whether your large congenital melanocytic nevus had nodules? *

| ○Yes |
| --- |
| ○No |

9. Did you ever feel physically painful of the large congenital melanocytic nevus? *

| ○Yes |
| --- |
| ○No |

10. Did you ever feel itching of the large congenital melanocytic nevus? *

| ○Yes |
| --- |
| ○No |

**Section III. Other Information Is Related to Large Congenital Melanocytic Nevus**

11. Did you ever have any neurological symptoms? *

| ○Yes, please clarify _________________ * |
| --- |
| ○No |

12. Did you have any other physical diseases? *

| ○Yes, please clarify _________________ * |
| --- |
| ○No |

13. Did your large congenital melanocytic nevus relapse after surgery? *

| ○Yes, please clarify in term of phenotype and time _________________ * |
| --- |
| ○No |

14. Did your large congenital melanocytic nevus become malignant? *

| ○Yes, please clarify in term of time, treatment and prognosis _________________ * |
| --- |
| ○No |

15. What was your surgical procedure? *

| ○Skin graft |
| --- |
| ○Hemorrhoidectomy |
| ○Flap graft |
| ○Skin dermabrasion |
| ○Surveillance |

16. Was your large congenital melanocytic nevus partially or entirely removed? *

| ○Partially |
| --- |
| ○Entirely |

17. Do you satisfy with your surgery outcome? *

| No | ○1 | ○2 | ○3 | ○4 | ○5 | Very Satisfied |
| --- | --- | --- | --- | --- | --- | --- |

That's all. Thank you for your cooperation.

If you have any further questions, please feel free to visit our clinic for consultation.

Signature of Interviewer: ;

Date and Time: ;
